# Preferences in adolescents and young people’s sexual and reproductive health services in Nigeria: a discrete choice experiment

**DOI:** 10.1186/s13561-024-00497-4

**Published:** 2024-03-22

**Authors:** Olujide Arije, Jason Madan, Tintswalo Hlungwani

**Affiliations:** 1 Institute of Public Health, Obafemi Awolowo University, Ile-Ife, Nigeria; 2 School of Public Health, University of Witwatersrand, Johannesburg, South Africa; 3 Warwick Medical School, University of Warwick, Warwick, UK

**Keywords:** Stated preferences, Discrete choice experiment, Adolescents and young people, Panel-data mixed logit model, Latent class logit model, Ogun State, Nigeria

## Abstract

**Background:**

Barriers to utilization of sexual and reproductive health (SRH) services by adolescents and young people (AYP) have persisted despite evidence that youth-friendly services have a positive effect on contraceptive use, and patient knowledge and satisfaction.

**Objective:**

The objective of this study was to elicit, and derive relative valuations for, attributes of SRH services that adolescents and young people value, and their willingness to pay for these services, in public health facilities.

**Methods:**

A discrete-choice-experiment (DCE) that was developed using a mixed methods approach was administered to AYP from Ogun State, Southwest Nigeria. The DCE attributes were: the type of staff; physical environment; health worker attitude; cost; waiting time; contraceptive availability; and opening hours. The choice tasks had two unlabeled alternatives and an opt-out option. Panel mixed logit choice model was used to fit the choice data, along with estimation of willingness to pay (WTP). Also, a latent class logit model was used to detect underlying preference heterogeneity among the respondents. Finally, the uptake of the services in health facilities was investigated by estimating the probabilities for selecting hypothetical health facilities under different scenarios.

**Results:**

A total of 859 AYP participated resulting in 6872 choice observations. The physical environment attribute had the highest utility rating relative to the other attributes, followed by preference for a doctor and for a service provider who was open and friendly. The cost and time coefficients were negative, revealing preference for lower cost and shorter waiting time. The latent class model had three classes that varied by their background characteristics. Probability of choosing any of the facility alternatives increased with introduction of more favorable facility characteristics.

**Conclusion:**

The pattern of preferences identified are potential targets for service design and delivery optimization that may result in improvements in service acceptability and utilization. These results strengthen the call for involving AYP in decision-making in health interventions for them and developing context-specific SRH programs for AYP in public health facilities.

**Supplementary Information:**

The online version contains supplementary material available at 10.1186/s13561-024-00497-4.

## Introduction

In Sub-Saharan Africa, some progress has been made in improving sexual and reproductive health (SRH) outcomes among adolescents and young people (AYP) aged 10–24 years. For instance, the adolescent fertility rate was reduced by more than 18% between 2000 and 2020, and the contraceptive prevalence rate among 15–24 years old increased by up to 10 percentage points between 1996 and 2015 [1]. However, much ground still remains to be covered. Available SRH services are often for maternal reproductive health, and those that are available are often not financially accessible [2]. Studies from Nigeria and South Africa show that many public health facilities are able to provide general health services but lack essential components for providing adolescent-specific services [3, 4]. Few adolescents know about the available SRH services, and fewer utilize them [5–8]. Barriers to the utilization of services include personal (e.g. AYP’s knowledge), cultural (community beliefs, stigma, caregiver lack of support), service-related (unprofessional attitudes of staff, insufficient staff skilled in adolescent sexual and reproductive health (ASRH), long waiting times, inconvenient operating time, and perceived lack of privacy and confidentiality), and structural (financial costs, distance to health facilities, and lack of supplies and medications) [4, 9–13]. In Nigeria, adolescents often do not have access to SRH services, experience stigmatization when using such services, and health workers are often judgmental [14, 15]; similar findings are reported in many sub-Saharan countries [16].

Inadvertently, barriers to services utilization have persisted despite evidence that shows that youth-friendly services have a positive effect on reducing teen pregnancy, contraceptive use, and patient knowledge and satisfaction [17]. Interventions are needed to address the SRH needs of AYP but they need to be built on the understanding of the preferences of AYP for SRH services. A systematic review of AYP SRH service utilization shows that health workers’ perceptions were different from AYP's perceptions [18]. The health workers perceived physical and financial barriers as fundamental while adolescents identified barriers emanating from providers’ attitudes as the key hindrance to their access and use of services. A cardinal goal in adolescent health services should be their participation in the design, implementation, and evaluation of health services for AYP. In the context of AYP, a positive experience of care is confidential, non-judgmental, non-discriminatory, participatory care [19]. The experience of care, as well as the quality of care (represented by service readiness) along with individual characteristics of the adolescents (knowledge, family setting, finances, etc.), are the likely determinants of user preferences in adolescent healthcare. The experience of care informs future use, hence preferences for services. Likewise, it is reasonable to assume that user preferences drive service utilization which loops back to inform the experience of care of users. [20].

It has been noted that quantitative preference assessments emphasize comparative benefit and provide quantitative evidence on the patient’s perceptions or experiences, perspectives, needs, and priorities regarding a disease, condition, treatment, service (including diagnostics and preventive services), or system [21]. Preference assessments can either be based on revealed or stated preferences [22]. Furthermore, revealed choices portray the world as it is because the alternatives from which to choose are directly observable and represent the market and personal limitations on decision making [23]. Stated preferences are different from actual choices. They are hypothetical and can include different options, but they don’t necessarily reflect real-world constraints or changes in the market. In the context of stated and revealed preferences, a trade-off can be defined as an exchange in which a participant gives up some amount of one attribute to gain more of another [24].

To improve the quality of care for adolescents and young people in ASRH services, it's important to understand their preferences. One way to do this is by investigating the different options available for ASRH services and examining the attributes of each option. By doing so, we can determine what combinations of attributes are most important to adolescents and young people, and use this information to prioritize the design of ASRH services. Ultimately, by using a preference-based approach to service design, we can ensure that ASRH services are tailored to the needs and preferences of adolescents and young people, thereby improving their quality of care. Discrete choice experiments have become the tool of choice to understand consumer behavior and preferences with essential applications in healthcare services [25]. This study aims to quantify the trade-offs adolescents and young people are willing to make between different attributes of ASRH services and their willingness to pay for their preferred services. It is hypothesized that AYP make trade-offs based on quality of care characteristics in their choice to uptake SRH services in public health facilities, and that they exhibit preference heterogeneity with respect to these services. By tailoring ASRH services to the needs and preferences of adolescents and young people, we can improve the quality of care and ensure that they receive the SRH services they need.

## Methodology

### Study setting

In this research, we set up a discrete choice experiment (DCE) to assess stated preferences of AYP in SRH services in public health facilities in Ogun State, Nigeria. In line with efforts to increase the uptake of SRH services by young people, the Ogun State government launched the Ogun State Adolescents and young people’s sexual and reproductive health strategic framework, 2018 – 2022 in 2019 [26]. Given the increasing adoption of adolescent health-focused policies at subnational levels, it was imperative to assess the conditions required for the most effective provision of SRH services for adolescents and young people. This study was carried out in Abeokuta-South Local Government Area (LGA), a predominantly urban LGA, and Ijebu-East, a predominantly rural LGA in Ogun State, Nigeria.

### Development of experimental design

This study involved the identification of attributes and attribute levels, development of the experimental design, piloting, data collection, and data analysis. We used a three-step process in developing the attributes and attribute levels for our study instrument. The first was to conduct a series of 16 focus group discussions (FGD) with AYP of between four and 10 participants each ensuring maximal variation (age group, sex, marital status, and location) [27]. The FGD included a process in which each participant was asked to list and rank the most important characteristics of optimal SRH services for AYPs. The lists were harmonized and the items were scored. The main (highest scoring) themes emerging from the harmonized priority list were converted into an initial set of attributes, and the subthemes as level. These initial attributes and levels were presented to a panel of methods and content experts in a virtual modified Delphi process to decide on the importance of the attributes in providing optimum SRH services for young people, and the appropriateness of the levels. The same set of attributes was presented to another set of AYP in a series of four FGD of between 8–10 participants each to clarify meanings, and test whether the wordings were well understood. The attribute development process is described in fuller details elsewhere [28].

### Experimental design

Ngene software [29] was used to create an initial D-efficient design for a multinomial logit model with zero priors which were used in a pilot test. The attributes were either dummy coded (type of staff, physical environment, staff attitude, contraceptives’ availability, open hours) and or continuous (cost of services, waiting time), the design was unlabeled, and there was an opt-out of foregoing the service To get an efficient experimental design, the size of the design matrix was defined by the number of choice tasks, |S| as shown below [30].

$$\left|S\right|=\frac{K}{\left|J\right|-1}$$

K is the total number of parameters to estimate, K (including label-specific constants and the coefficients of the dummy coded attributes), and J is the number of alternatives. In this study, K = 12, and J = 3, therefore the minimum number of choice sets was ≥ 6. The syntax used for the design in NGene specified 24 rows and 3 blocks with an efficiency criterion of a multinomial logit model that minimizes the D-error. The algorithm used was Mfederov, which relaxes the attribute level balance constraint. In addition, the priors used for the model were set to be uninformative, i.e., near-zero values for each attribute, with some variation across the different levels of the attributes. The syntax also included options to check for dominance of any of the specified alternatives. The presented choice tasks included an unforced choice between two health facility alternatives, as well as the option to opt-out (neither). If respondents chose to opt-out, they were then presented with a forced choice.

### Pilot testing and survey amendment

**Fig. 1:**
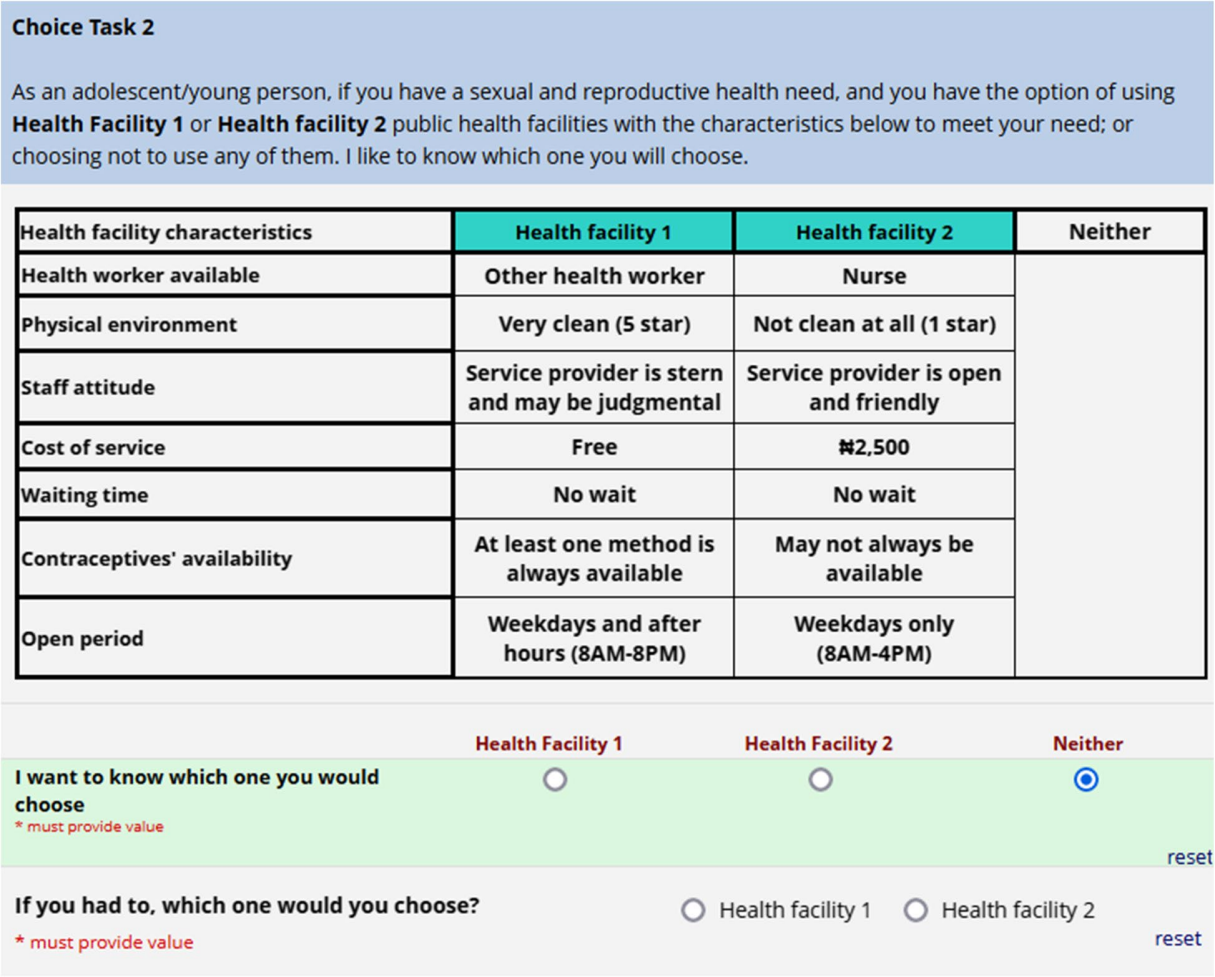
Sample of choice task

The DCE tool was pilot-tested with a convenience sample of 69 AYPs who completed one block of eight choice tasks each. To generate the final experimental design, we utilized the data from the pilot study to estimate prior parameters, employing a multinomial logit model. Additionally, based on the findings from the pilot study, we made further revisions to the DCE questionnaire. For our final experimental design, we used a Bayesian efficient design that reduces the statistically required number of profiles down to a reasonable and manageable number [24]. The syntax used for the final experimental design was similar to the one used in the pilot design with important additions. The design used quasi-random Sobol draws (200 draws) for the Bayesian priors. Each attribute was assigned a coefficient value, which was determined using near-zero Bayesian priors to estimate the posterior distribution of the parameters. The near-zero prior used for the coefficient values indicated that the ranking order of the attributes was known but there was uncertainty about their relative importance. This approach helped to reduce the uncertainty in the model’s estimated parameters and improve the design’s efficiency. The final design had two unlabeled alternatives and an opt-out option, and seven attributes with two to four levels each (Table 1). While all other attributes and levels are self-explanatory, we categorized health facility cleanliness into three distinct levels: ‘Very clean’, ‘Moderately clean’, and ‘Not clean at all’. These cleanliness levels were assessed using a star rating system. ‘Very clean’, denoted by a 5-star rating, represents an environment that is pristine and exceptionally well-maintained, meeting the highest hygiene standards. ‘Moderately clean’, corresponding to a 3-star rating, indicates a reasonably clean environment that meets acceptable hygiene standards. Finally, ‘Not clean at all’, associated with a 1-star rating, signifies an environment that falls significantly short of basic hygiene standards. A sample choice task is provided in Fig. 1. In the final questionnaire, a choice task with a dominant choice set was included as part of the validity check.

**Table 1:** Attributes and levels of the DCE

Attributes	Definition	Levels
Type of staff	These are the different types of health workers young people are likely to meet with at the health facility	• Doctor • Nurse • Other health worker
Physical environment	This is a rating of how clean the physical environment of the health facility is as perceived by the adolescent and young person	• Very clean (5 star) • Moderately clean (3 star) • Not clean at all (1 star)
Staff attitude	This attribute is about how the adolescents and young people perceive the behaviour of the health workers in the health facility towards them	• Service provider is open and friendly • Service provider is stern and may be judgmental
Cost of services	This is the total amount the adolescent or young person is willing to spend for all services obtained in the health facility	• Free • ₦500 • ₦1500 • ₦2500
Waiting time	This is the amount of time that the adolescent or young person has to wait before being attended to in the health facility	• No wait • 30 min • 60 min
Contraceptives’ availability	This attribute is about availability of contraceptives to the adolescents and young people that need them anytime they go to the health facility	• At least one method is always available • May not always be available
Open hours	This is the period during which services can be accessed by adolescents and young persons	• Weekdays only (8AM—4PM) • Weekdays and after hours (8AM—8PM) • Weekdays and weekends

### Sample size

Following pilot testing from which ‘prior parameter’ values (i.e. values derived by Bayesian probability that would express researcher’s belief about quantity of the parameter before empirical measurement) can be generated, the sample size formula proposed by de Bekker et al. [31] was used to calculate an ideal sample size, which allows for sufficient power for hypothesis testing. The formula is:

$$N >{\left(\frac{\left({Z}_{1-\beta }+{Z}_{1-\alpha }\right)\sqrt{{\Sigma }_{\gamma n}}}{\delta }\right)}^{2}$$

Where N is the required minimum sample size, α is the significance level, 1-β is the statistical power, δ is the effect size derived from a pilot study and ${\Sigma }_{\gamma }$ is the asymptotic variance–covariance (AVC) matrix for the nth diagonal element. Using the algorithm provided by de Bekker et al. [31] we obtained sample sizes for each of the parameters in the DCE model to be estimated. Given that logistical exegesis would not permit for sample size as large as the maximum we estimated, we used a next plausible sample size of 859.

### Sampling technique

A multi-stage sampling technique was employed in three stages. For each LGA, the list of political wards and the estimated population were acquired, and five of the largest wards were selected per LGA by ranking their population in the first stage. The ranking was done to support the logistics of the study such that there would be easier access to the study population. In each chosen ward, ten communities were selected by simple random sampling in the second stage. Starting from a randomly selected building in each street/community chosen, one household with a person aged 15 – 24 years, was selected in every alternate building until the sample size assigned to that community was exhausted. In each selected household, a person aged 15 – 24 years was interviewed. In household with more than one person aged 15 – 24 years, the interviewee was selected by balloting. Equal sample sizes were assigned to each community. To ensure equal representation of the male and female population alternative wards were assigned to male and female participants. Also, we assigned participants in alternate wards to respond to alternate blocks of choice tasks.

### Data collection

The final survey was carried out in August 2022. The DCE questionnaire, including clear instructions, were provided to respondents, including a sample question/choice scenario. Elicitation of preference was by face-to-face interviews during the survey. Participants completed the DCE choice questions on a tablet device but administered by an interviewer. The interviewers facilitated completion by reading out as well as showing each choice task (clarifying any ambiguity); no respondents reported finding the DCE difficult to complete. In addition to the choice tasks that participants were requested to complete, they also responded to questions on sociodemographic characteristics (location, age, sex, occupation, educational status, marital status, religion, ethnicity, socio-economic status) and, SRH behavior (utilization of health services, use of contraceptives and history of sexually transmitted infection).

### Data analysis

We analyzed the final survey data using the choice modeling module of Stata 16 [32]. There were no missing data. We presented the respondents’ characteristics using descriptive statistics. We first fitted a multinomial logit (MNL) model for our data (not reported). Subsequently, we used the panel mixed logit choice model that fits a logit model to choice data in which decision-makers make repeated choices, producing a structure typical of panel data. Panel-data mixed logit models use random coefficients to model the correlation of choices across alternatives in order to take preference heterogeneity into account, by the assumption that preferences in different attributes are independent [33, 34]. The Panel-data mixed logit model had a similar fit to the MNL model, therefore we decided to retain Panel-data mixed logit model in keeping with the panel structure of the data. Our basic estimation models specified the probability that a respondent will choose one of the health facilities or none. Our analysis model is based on the random utility theory in which the utility that can obtained from consuming a product/service has deterministic and random components. The deterministic component depends on the vector of attributes of the product/service and their marginal utilities. The random component is the error term in the regression specification which is composed of an individual specific random effect (because of the panel nature of the data) and a second independent error term that has an extreme value type one distribution. The latter allows for the estimation of the parameters as a logit model. The deterministic part of the utility is thus assumed to be linear and is expressed in the following simplified function:

$${V}_{j}\hspace{0.17em}=\hspace{0.17em}{\beta}_{0}\hspace{0.17em}+\hspace{0.17em}{\beta}_{1}(staff\_doctor)\hspace{0.17em}+\hspace{0.17em}{\beta}_{2}(staff\_nurse)\hspace{0.17em}+\hspace{0.17em}{\beta}_{3}(environment\_Mod.clean)\hspace{0.17em}+\hspace{0.17em}{\beta}_{4}(environment\_Very\,clean)\hspace{0.17em}+\hspace{0.17em}{\beta}_{5}(Cost)\hspace{0.17em}+\hspace{0.17em}{\beta}_{6}(Time)\hspace{0.17em}+\hspace{0.17em}{\beta}_{7}(Attitude)\hspace{0.17em}+\hspace{0.17em}{\beta}_{8}(FP\_One\,method)\hspace{0.17em}+\hspace{0.17em}{\beta}_{9}(Opening\,time\_Weekdays\,\&\,Weekends)\hspace{0.17em}+\hspace{0.17em}{\beta }_{11}(Opening\,time\_Week\,days\,\&\,after\,hours)$$

Where V_j_ is the utility of choosing an option j, the corresponding β_1_ – β_7_ are the part-worth utility attached to attribute levels in a preference space model. All design attributes were treated as categorical and dummy coded (the level with the lowest expected utility were made the reference category), apart from cost and waiting time which were treated as numerical. Additionally, all attributes were treated as normally distributed random parameters with no assumed correlations between attributes. There were no alternative specific variables in the data, therefore, all the design attributes were specified as fixed, including the alternative specific constants (ASC). This also facilitated the estimation of willingness to pay (WTP). Cost was presented to participants in Naira but for the analysis we converted to Dollars using an exchange rate of $1 to ₦420. This rate was the average central exchange rate in the year of the study (2022) according to the Central Bank of Nigeria (https://www.cbn.gov.ng/rates/ExchRateByCurrency.asp).

We specified a model in which we included respondents’ characteristics as case-specific variables. The variables that we used were location (Rural/Urban), educational status (None/Primary/Secondary/Tertiary), perceived socioeconomic standing (Lower/Middle/Higher), and sexual behavior (Never had sex/Used contraceptive in last sex/Didn’t use contraceptives during last sex). Perceived socioeconomic standing was assessed by asking the participants to rate themselves on a scale of 1 – 10 on how they compare themselves to the most affluent/wealthy people in their society and their responses were categorised into terciles. The choice of the case-specific variables was based on those respondents’ characteristics that were statistically significant in a bivariate association with the choice variable (See Supplementary document 1, Table 1 and 2). Model fit was assessed using both a log-likelihood ratio (LLR) test and Akaike Information Criterion (AIC). The model results were separately specified as odds ratios and we also estimated WTP for each attribute/level as the ratio of the marginal utility of each attribute/level to its marginal ‘disutility’ (negative utility) of cost. We did this by estimating the ratio of the attribute and price following the mixed logit model specification.

**Table 2:** Background characteristics of study participants

Background characteristics	Freq. (%) *N* = 859
**Local Government Area**	
Urban LGA	432 (50.3)
Rural LGA	427 (49.7)
**Sex**	
Female	430 (50.1)
Male	429 (49.9)
**Age group**	
15–19 years	589 (68.6)
20–24 years	270 (31.4)
**Occupation**	
Farming	11 (1.3)
Student	460 (53.6)
Apprentice/Artisan	266 (31.0)
Self-employed/Trading	61 (7.1)
Unemployed	20 (2.3)
Others	41 (4.8)
**Educational status**	
None	27 (3.1)
Primary	104 (12.1)
Secondary	682 (79.4)
Higher	46 (5.4)
**Marital status**	
Married	54 (6.3)
Divorced/Separated	4 (0.5)
Never married	801 (93.2)
**Religion**	
Islam	292 (34)
Orthodox (Non-Catholic Christian)	147 (17.1)
Catholic Christian	52 (6.1)
Pentecostal Christian	336 (39.1)
Others	32 (3.7)
**Ethnicity**	
Igbo	53 (6.2)
Yoruba	758 (88.2)
Others	48 (5.6)
**Subjective socioeconomic standing (terciles)**	
Lower	324 (37.7)
Middle	387 (45.1)
Higher	148 (17.2)
**Ever used primary or secondary health facility**	
No	355 (41.3)
Yes	504 (58.7)
**Sexual behavior**	
Never had sex	130 (15.1)
Used contraceptive in last sexual encounter	214 (24.9)
Didn’t use contraceptive in last sexual encounter	515 (60.0)
**Ever had STI**	
No	784 (91.3)
Yes	75 (8.7)

Furthermore, we carried out a latent class analysis to detect any underlying preference heterogeneity among the respondents. Latent Class Analysis (LCA) is a statistical method for identifying unmeasured class membership among subjects using either categorical or continuous observed variables [35]. The latent class conditional logit models use optimization methods for maximum likelihood estimation. Models with increasing numbers of classes were fitted and compared based on convergence and Bayesian Information Criteria (BIC). Using our final model, we assigned each participant to a class by predicting the posterior probabilities that the participant is in a particular class, considering his or her sequence of choices. We assigned participants to a class based on their highest probability of being a member. Then, we assessed the characteristics of the group by cross-tabulating group membership with the selected participants’ characteristics. We also assessed willingness to pay based on class membership from the latent class logit model.

Finally, we investigated the uptake of the services in health facilities by calculating the probabilities for selecting those hypothetical health facilities under different scenarios using the result from the panel-data mixed logit model. We conducted a simulation involving eight scenarios. For four of these simulations, both Alternative 1 and Alternative 2 were associated with a cost of $10 and a waiting time of 2 h. In the best-case scenario for both Alternative 1 and Alternative 2, patients could anticipate receiving care from a doctor in a very clean (5-star) environment. The service provider was characterized as welcoming and approachable, with no additional costs or waiting times. Moreover, at least one method of contraception was consistently available, including on weekdays and after hours. This configuration was applied separately to Alternatives 1 and 2, accounting for two scenarios. Conversely, the worst-case scenario for both Alternative 1 and Alternative 2 involved healthcare providers other than nurses and doctors. The healthcare facility received the lowest cleanliness rating (1 star), and the service provider was portrayed as stern and possibly judgmental. Costs was the most expensive, waiting time could be up to one hour, and there was no assurance of the availability of contraceptives. This scenario was also separately applied to Alternatives 1 and 2, totaling two scenarios.

The probability of choosing any of the alternatives is given by the equation:

$${P}_{i1}=\frac{{e}^{{V}_{1}}}{{\sum }_{j\in J}{e}^{{V}_{j}}}$$

Where *P*_*i1*_ is the probability that alternative 1 will be chosen among *J* alternatives, *V*_*1*_ is the utility associated with alternative 1, *V*_*j*_ is the utility associated with each of the J alternatives. We simulated the choice of participants, and predicted the probability of choice using the estimates from our mixed logit preference space model. Subsequently, we generated confidence intervals by running 1000 simulated coefficient vectors using the estimated betas and their variances.

We have included as supplementary material (see Supplementary document 2) to this study a DCE studies quality checklist based on criteria/checklist by Bridges et al. [33], Hauber et al. [36], Mandeville et al. [37], and Lancsar & Louviere [38].

## Results

### Background characteristics

A summary of respondent characteristics is provided in Table 2. Of the 859 respondents, more than half were from the urban LGA (50.3%) and were female (50.1%). The majority were in the 15–19 years’ age group (68.6%), were students (53.6%), had completed secondary school education (79.4%), and were of Yoruba ethnicity (88.2%). The highest proportion was among Pentecostal Christians (39.1%), and in the middle class for subjective socioeconomic standing (45.1%). The highest proportion of participants had ever used the services of a primary or secondary public health facility (60.0%). Among all participants, 84.9% were sexually active, and 8.7% had ever had a sexually transmitted infection. Although the choice of the opt out alternative was only 4.2% in our study, we retained it in our analysis because the option represents a scenario the study population would ordinarily face in reality. Here, we present the results for the unforced data only.

### Mixed logit (MXL) model

Data from participants yielded 6872 observations (8 choice scenarios each by 859 participants) and participants selected one of the two health facilities 95.8% of times. All the attributes influenced respondents’ preferences significantly apart from the opening hours of the facilities (Table 3). All standard errors are cluster-robust, which allows for arbitrary correlation between the disturbance terms at the individual level. Participants had 48.4% and 66.1% higher odds of preferring a Moderately clean (3 star) and Very clean (5 stars) health facility, respectively, over a health facility rated as Not clean at all (1 star). Similarly, they had 53.6% and 35.8% higher odds of preferring a doctor or a nurse respectively over other health workers. Preference for a service provider who was open and friendly was associated with 25.1% higher odds than one who was stern and might be judgmental. The cost and time coefficients were negative, confirming that participants preferred services with a lower cost and shorter waiting time. However, there was only a 3% lower odds of choosing a service given an increase in cost by $1 (at the time of the survey in August 2022, $1≈N420), and a 15% lower odds of choosing a service given an additional waiting hour. Furthermore, participants had a 16.9% higher odds of preferring a facility where availability of at least one method is always guaranteed compared to uncertainty about availability of a contraceptive method on the day of visit.

**Table 3:** Mixed Logit choice model

Variables	β	Robust SE	*p*-value	OR	WTP
**Type of Staff**					
Other health worker^a^	0.000	-	-	1.000	0.000
Doctor	0.429	0.048	< 0.001	1.536	14.143
Nurse	0.306	0.036	< 0.001	1.358	10.076
**Environment**					
Not clean at all (1 star)^a^	0.000	-	-	1.000	0.000
Moderately clean (3 star)	0.394	0.040	< 0.001	1.484	13.001
Very clean (5 star)	0.508	0.045	< 0.001	1.661	16.730
**Health worker attitude**					
Service provider is stern and may be judgmental^a^	0.000	-	-	1.000	0.000
Service provider is open and friendly	0.224	0.030	< 0.001	1.251	7.386
**Cost**	-0.030	0.007	< 0.001	0.970	-
**Time**	-0.160	0.027	< 0.001	0.852	-5.260
**Contraceptive**					
May not always be available^a^	0.000	-	-	1.000	0.000
At least one method is always available	0.156	0.030	< 0.001	1.169	5.144
**Opening hours**					
Weekdays only^a^	0.000	-	-	1.000	0.000
Weekdays and weekends	0.033	0.038	0.387	1.034	1.093
Weekdays and after hours	-0.002	0.037	0.967	0.998	-0.051
**Health facility ASC**					
Neither^a^	0.00	-	-	1.000	-
Health Facility 1	1.949	0.093	< 0.001	7.021	-
Health Facility 2	1.773	0.095	< 0.001	5.890	-

*SE* Standard error*, OR* Odds ratio*, WTP* Willingness to pay, *ASC* Alternative specific constant

^a^*Reference category*

Corresponding to the odds of preferences reported above, participants were willing to pay an additional $14.1 and $10.0 to see a doctor and a nurse respectively over being attended to by other types of health workers. Similarly, they were willing to pay an additional $16.7 to visit a very clean health facility over one that was not clean and this was more important than staff qualification. The alternative specific constant (ASC) for both Health facility 1 and 2 were positive and statistically significant showing that respondents positively valued utilizing the health facility compared to opting out.

### Latent class modelling

We specified a three-class model based on membership of selected case-specific variable categories to account for the sequence of choices because the model did not achieve convergence beyond four classes, and the three-class model had the lowest Bayesian information criterion (BIC) of the three models that achieved convergence (Supplementary document 1, Table 3). In the three-class model, participants in Class 1 had a statistically significant higher preference utility for a friendly and open service provider (β = 1.430, *p* < 0.001) while those in Class 2 had a lower preference utility (β = -0.382, *p* = 0.007) (Table 4). Participants in Class 3 were the most cost and waiting time averse with a cost preference utility of -0.125 (*p* < 0.001) and time preference utility of -0.193 (*p* = 0.002). Participants in Class 2 were the least cost averse with a cost preference utility of 0.090 (*p* < 0.001). Participants in the three classes had an almost equal preference for facilities that have contraceptives (0.230 – 0.296) and all were statistically significant. Participants in Class 3 had the least preference utility for the opening hours of the health facility (weekdays and after hours: β = -0.312, *p* < 0.001).

**Table 4:** Latent Class conditional logit model

Attributes and levels	Class 1			Class 2			Class 3		
	**β**	**SE**	***p***	**β**	**SE**	***p***	**β**	**SE**	***p***
**Type of Staff**									
Other health worker^b^									
Doctor	0.022	0.174	0.900	1.948	0.352	< 0.001	-0.042	0.105	0.687
Nurse	0.456	0.176	0.010	0.831	0.191	< 0.001	0.216	0.076	0.005
**Environment**									
Not clean at all (1 star)^b^									
Moderately clean (3 star)	1.584	0.239	< 0.001	0.536	0.128	< 0.001	-0.105	0.108	0.329
Very clean (5 star)	1.675	0.212	< 0.001	0.611	0.105	< 0.001	-0.114	0.126	0.364
**Health worker attitude**									
Service provider is stern and may be judgmental^b^									
Service provider is open and friendly	1.430	0.245	< 0.001	-0.382	0.140	0.007	0.094	0.064	0.143
**Cost**	-0.046	0.030	0.128	0.090	0.022	< 0.001	-0.125	0.023	< 0.001
**Time**	-0.123	0.114	0.278	-0.163	0.088	0.062	-0.193	0.062	0.002
**Contraceptive**									
May not always be available^b^									
At least one method is always available	0.296	0.136	0.029	0.235	0.114	0.039	0.230	0.067	0.001
**Opening hours**									
Weekdays only									
Weekdays and weekends	0.328	0.142	0.021	-0.047	0.119	0.690	-0.150	0.096	0.120
Weekdays and after hours	0.817	0.234	< 0.001	0.051	0.132	0.698	-0.312	0.087	< 0.001
**Health facility**									
Neither^b^									
Health Facility 1	3.046	0.232	< 0.001	3.495	0.205	< 0.001	3.383	0.203	< 0.001
Health Facility 2	2.878	0.212	< 0.001	3.579	0.220	< 0.001	3.062	0.208	< 0.001
**Participant characteristics**	**Share 1**			**Share 2**^a^			**Share 3**		
Rural^c^	0.093	0.263	0.725				0.096	0.251	0.702
Apprentice/Artisans^d^	0.234	0.307	0.446				0.400	0.280	0.153
Other occupations^d^	-0.003	0.390	0.994				0.082	0.330	0.803
Primary education^e^	0.873	1.298	0.501				-0.224	0.761	0.769
Secondary education^e^	0.458	1.273	0.719				-0.839	0.702	0.232
Tertiary education^e^	1.271	1.360	0.350				-2.006	1.072	0.061
Middle SES^f^	0.350	0.326	0.284				-0.640	0.239	0.007
Higher SES^f^	1.289	0.400	0.001				-0.061	0.355	0.864
Used contraceptive ^g^	-0.458	0.314	0.145				-0.372	0.272	0.171
Never had sex ^g^	-0.536	0.381	0.159				0.123	0.308	0.689
Constant	-1.164	1.354	0.390				1.257	0.851	0.140

*SES* Perceived Socioeconomic status

^a^Reference class

^b^Reference category; Reference categories for ‘Participant characteristics’

^c^Urban

^d^Student

^e^No formal education

^f^Lower SES

^g^Didn’t use contraceptive in last sexual act

**Fig. 2:**
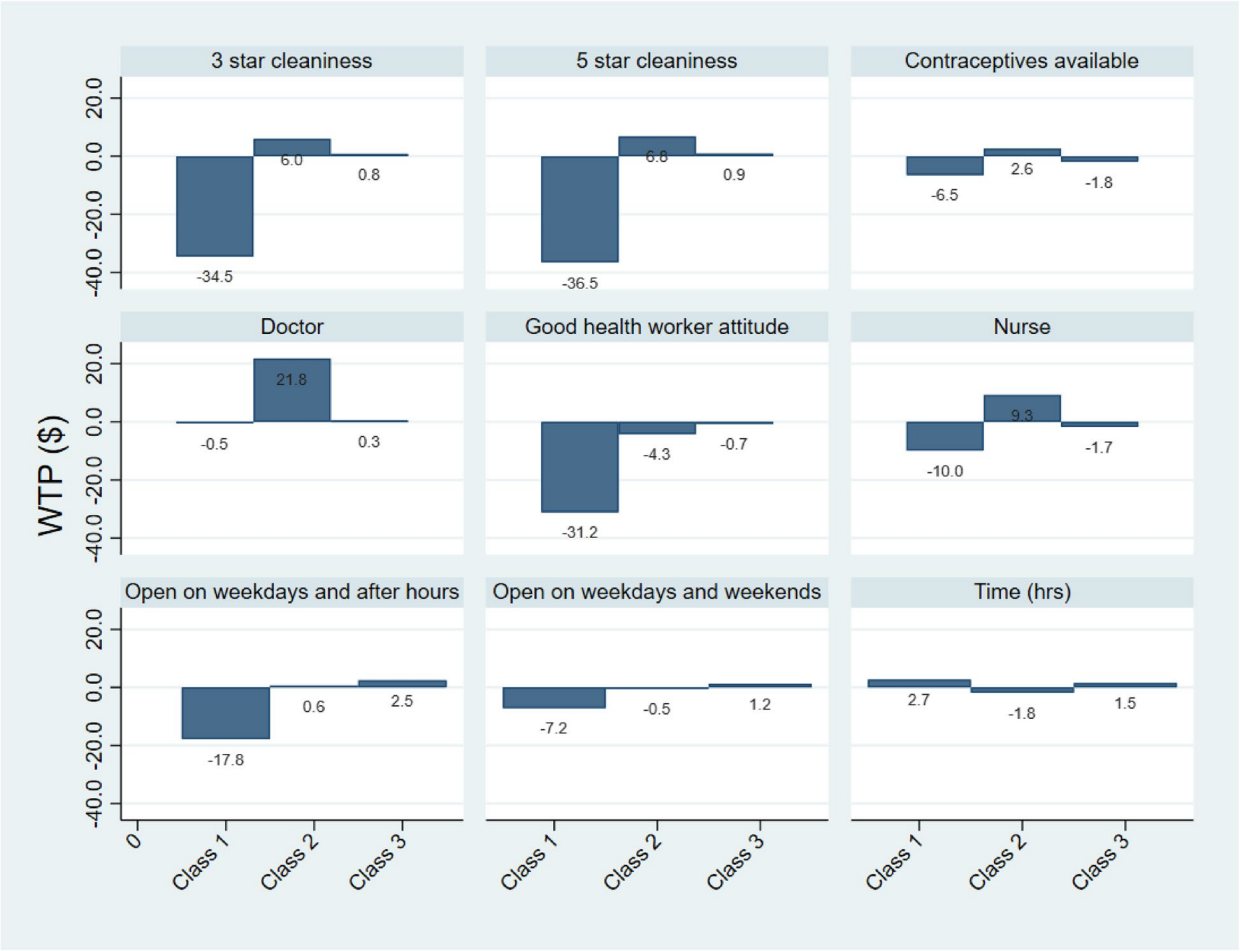
Bar charts showing pattern of willingness to pay across the three latent classes

The pattern of preference already described is also seen in the willingness to pay preference space with participants in different classes deferring by their willingness to pay (Fig. 2). The coefficient (i.e. y-axis) in each class represents how much more (or less, in dollars) each person in that class is willing to pay to have that level of the attribute, compared to the reference level, or by one unit change in the relevant attribute.

As shown in Table 5, the participants assigned to Class 1 were 206 (24.0%), Class 2, 276 (32.1%), and Class 3, 377 (43.9%). Cross tabulation revealed that class membership was statistically significantly associated with participants’ location, educational status, perceived socioeconomic standing, and sexual behavior (Table 5).

**Table 5:** Association between latent class membership and selected participant characteristics

Participant characteristics	Class 1	Class 2	Class 3	Test of association
	No. (%)	No. (%)	No. (%)	
**Local Government Area**				χ(2) = 8.1464 ***p* = 0.017**
Abeokuta South LGA	120 (27.8)	139 (32.2)	173 (40.0)	
Ijebu East LGA	86 (20.1)	137 (32.1)	204 (47.8)	
**Occupation**				χ(4) = 8.2687 *p* = 0.082
Student	120 (26.1)	158 (34.3)	182 (39.6)	
Apprentice/Artisan	59 (22.2)	75 (28.2)	132 (49.6)	
Others	27 (20.3)	43 (32.3)	63 (47.4)	
**Educational status**				χ(6) = 65.6113 ***p* < 0.001**
None	2 (7.4)	6 (22.2)	19 (70.4)	
Primary	18 (17.3)	22 (21.2)	64 (61.5)	
Secondary	157 (23.0)	235 (34.5)	290 (42.5)	
Higher	29 (63.0)	13 (28.3)	4 (8.7)	
**Subj socioeconomic standing**				χ(4) = 100.0104 ***p* < 0.001**
Lower	35 (10.8)	92 (28.4)	197 (60.8)	
Middle	103 (26.6)	151 (39.0)	133 (34.4)	
Higher	68 (45.9)	33 (22.3)	47 (31.8)	
**Sexual history**				χ(4) = 9.0371 *p* = 0.060
Didn't use FP	139 (27.0)	156 (30.3)	220 (42.7)	
Used FP	46 (21.5)	77 (36.0)	91 (42.5)	
Never had sex	21 (16.2)	43 (33.1)	66 (50.8)	
**Total**	**206 (24.0)**	**276 (32.1)**	**377 (43.9)**	

### Predictive analysis

We simulated a number of scenarios to assess changes in utilities under different scenarios (Table 6). For the scenario in which we increased the cost of services to $10 in Health facility 1 the probability of choosing Health facility 1 decreased from the baseline while the probability of choosing Health facility 2 increased from the baseline. There was only a minimal increase in the probability of choosing the opt-out option. The converse was the case when the cost of services was increased to $10 in Health facility 2. Similarly, when waiting time was increased to two hours in Health facility 1, the probability of choosing the facility decreased from baseline, and so was the case when waiting time was increased in Health facility 2. We simulated a scenario in which the most favorable levels were assigned to Health facility 1 and then to Health facility 2. The probability of choosing the facilities in the two scenarios was much increased in both facilities. These findings are similar to the distribution of responses to the validation choice task in which all the best levels were assigned to health facility 1.

**Table 6:** Probabilities of selecting alternatives for different simulated scenarios

Scenarios	Health Facility 1	Health Facility 2	Neither
	**Probability (CI)**	**Probability (CI)**	**Probability (CI)**
**Base uptake**	0.521 (0.511 – 0.532)	0.437 (0.427 – 0.448)	0.420 (0.360 – 0.4.70)
**Cost = $10, alternative 1**	0.429 (0.396 – 0.468)	0.488 (0.459 – 0.514)	0.083 (0.067 – 0.100)
**Time = 2 h, alternative 1**	0.425 (0.396 – 0.454)	0.491 (0.466 – 0.517)	0.083 (0.069 – 0.101)
**Cost = $10, alternative 2**	0.568 (0.534 – 0.597)	0.352 (0.316 – 0.393)	0.081 (0.067 – 0.097)
**Time = 2 h, alternative 2**	0.571 (0.546 – 0.593)	0.348 (0.322 – 0.376)	0.081 (0.068 – 0.098)
**Best scenario (alternative 1)**	0.797 (0.768 – 0.823)	0.173 (0.150 – 0.200)	0.029 (0.025 – 0.035)
**Best scenario (alternative 2)**	0.228 (0.199 – 0.259)	0.739 (0.705 – 0.770)	0.033 (0.028 – 0.039)
**Worst scenario (alternative 2)**	0.575 (0.551 – 0.596)	0.343 (0.316 – 0.371)	0.082 (0.068 – 0.099)
**Worst scenario (alternative 1)**	0.420 (0.395 – 0.446)	0.496 (0.476 – 0.512)	0.084 (0.070 – 0.102)
**Validation choice task**	0.744 (0.738 – 0.750)	0.241 (23.5 – 0.247)	0.150 (0.140 – 0.170)

*CI* Confidence interval

## Discussions and conclusion

### Discussions

To sustain the policy drive to mainstream SRH services for AYP in Nigeria, robust preference elicitation approaches such as DCE that will allow policy makers to focus on improvements that matter to users are useful in program design. There is a paucity of documented evidence in the literature that the DCE method has been used in the context of ASRH research in Nigeria. This study, therefore, offers an opportunity in participatory research for designing adolescent health services as it allows for giving voice to AYP by recognizing and quantifying their priorities and preferences in SRH services. Baseline preference in this study shows that AYP are more likely to opt for rather than forego SRH care. In 2019, a study in Zambia reported that young people preferred to receive reproductive health services in community-based centers and not the health facility [39]. Findings from the qualitative research done for the development of the attributes and levels for this study indicated that the preferred source of sexually transmitted infection (STI) services included local herb sellers and traditional birth attendants [27]. Other studies in Nigeria showed that less than half of the participants used health facilities as the source of their family planning services [40, 41]. Studies from other developing countries have indicated reasons such as poor awareness, inadequate privacy, insufficiency of medicines and supplies, cost, and inconvenient operating hours as reasons for not using the health facilities among AYP [6, 16, 42, 43]. In all these cases, it is likely that the reason for use of alternative SRH care is because the facilities are not offering the package of care that AYP find suitable, and not that they do not like to use health facilities [44]. This current study demonstrates that AYP are more likely to use services when their preferences for services are addressed.

The physical environment of the health facilities was the most important attribute for our study participants overall. Though Ullan et al. did their work in a developed country setting, their study showed significant differences between adolescents’ and adults’ preferences for hospital design [45]. While no such comparison was done in this work, our findings lay credence to the need to be deliberate in the design and maintenance of health facilities to encourage the utilization of the services by AYP.

Participants preferred being attended to by doctors than nurses, and by nurses than by other cadres of health workers. This may be related to a need to be sure that the health worker is truly competent. In contrast, a DCE study conducted in Malawi suggested that youths do not categorically prefer a particular type of service provider (i.e., government facility, private facility, outreach service, or community-based services) [46]. This is of importance because it indicates that it is perhaps the other characteristics of the services that matter, and not the location for providing them. In any case, we can infer from this study that if the public health facilities have the right characteristics, AYP will utilize them.

Furthermore, other studies have also reported that confidentiality and non-judgmental attitude of health workers are some of the factors that drive preference for and utilization of facility-based health services [47–50]. Participants in this study also placed a high premium on health workers that are friendly and open. The qualitative research we did for the development of the attributes of this study also clearly indicated this [27]. As would be expected, time and cost were important attributes in the preferences of the study participants; they preferred shorter waiting times and less costly services. Estimates of willingness-to-pay show that AYP would be willing to pay about $5 for a reduction in waiting time by one hour. However, this should be interpreted with caution as a willingness to pay does not necessarily translate to the ability to pay. They may not even have access to financial resources to make the payment, as the majority of the participants considered themselves to be in the middle or lower subjective socioeconomic class. Drawing policy conclusions or pricing strategies based on estimates of WTP may therefore prove problematic.

Also, the willingness to pay estimates in both the mixed logit and latent class logit models are outside the range of costs presented to the participants, given the exchange rate used in this study. This raises concerns regarding attribute attendance and suggests that participants may have ignored the cost attribute. Attribute non-attendance refers to a situation where participants do not consider an attribute when making their choices [51]. Non-attendance may occur for various reasons, such as attribute complexity, lack of understanding, or lack of relevance to participants. In our study, the negative coefficient on cost suggests that participants preferred lower cost. However, the cost range used may not have been reflective of the actual costs that participants face in their daily lives. This could have led to non-attendance of the cost attribute as participants may not have considered the cost levels presented as realistic. Non-attendance of an attribute can impact the validity of the DCE results. Nevertheless, it is not uncommon for participants to attend to some attributes more than others or ignore some attributes entirely in a DCE. While the issue of non-attendance of the cost attribute is a valid concern, we still believe that our results are informative and can be used to enhance the design and delivery of adolescents and young people’s sexual and reproductive health services in Nigeria. Future research could explore the reasons for non-attendance of the cost attribute and investigate whether similar findings are observed in other settings or among other populations.

Across the latent classes, there was no significant difference in the preference for the availability of contraceptives. This may be an indication of a negative attitude of AYP to the use of contraceptives based on poor knowledge [13], perceptions that they are meant for the married [52], and impact on future fertility [53]. This study also showed that urban young people have different preferences from rural young people. Similarly in other studies, determinants of access and use of reproductive health services by adolescents include education and socio-economic status [54, 55]. As such, programs designed for AYP need to put this into consideration.

The other study we found focused on the preferences of young people for HIV testing services [56], which we could not directly compare with our study that has a broader focus. The work of Micheals-Igbokwe et al. [25, 46, 57] show that young people preferred to use family planning services if they needed it than opting not to. Their participants were twice as likely to prefer a friendly service provider. Additionally, the participants in their study had a preference for lower cost of services as reported in the current study. In contrast, a reliable supply of contraceptives was positively valued, and waiting time was not a very important influence on the choices of participants. In our study, we did not find statistically significant differences in preference by age and sex, or history of utilization of services. Similarly, Micheals-Igbokwe et al. [25] could not categorically conclude that different age categories had different preferences because the differences in preferences were not systematic enough across the service attributes.

The predictive analysis we did indicated that indeed AYP are generally sensitive to time and cost. The prediction also mimicked the validation choice question we included in which much more persons opted for the alternative that had the lowest cost and waiting time. The results of the predictions also illustrate that increasing prices of services or longer waiting time would increase the proportion who opt out of the services. When health facility 1 had the best scenario, there was a higher probability of selecting it compared to when health facility 2 had the best scenario. Also, when health facility 1 had the worst scenario, it had a higher probability of being selected compared to when health facility 2 had the worst scenario. This indicates that respondents are more likely to choose the leftmost scenario in an experimental design that reads from left to right. Our findings underline the need to include alternative specific constants analysis models even when experiments are unlabeled in order to be able to account for unobservables such as left–right bias [30].

### Limitations

DCE data are largely hypothetical and may not mirror real-world choices, Also, the estimates provided in our study are an indication of the relative value or importance of attributes to respondents, but they may not predict future behavior or health outcomes. However, they help to highlight nuances that would otherwise have been missed. The willingness to pay estimates may not correspond to the actual amount participants are willing to pay. Also, for the prediction analysis, predicting the choice of health facility beyond the study sample requires further recalibration. Even then with the use of unlabeled alternatives, the actual choice of any of Facility 1 or 2 is of no practical use. However, the relative differences in the WTP and probabilities of choosing an alternative help to illustrate the trade-off that AYP make in their choice of SRH services.

### Policy recommendations

This study has demonstrated one of the ways of giving a voice to adolescents in the design of health services that concern them. It established that when the preferences of adolescents are available in a public health facility, they are more likely to use the facility than not. However, it is necessary to contextualize interventions that target AYP, and not use one-size-fits-all approach [58]. Such contextualization requires basic research to characterize the young people being targeted at any time. However not every intervention will be able to sponsor primary research. We call for longitudinal studies in Nigeria and African settings that will make data available for secondary analysis on an ongoing basis. An example can be drawn from the National Longitudinal Study of Adolescent to Adult Health (Add Health) in the United States of America [59] which has been ongoing since 1994.

Concerning the cadre of health workers at public health facilities, it is impractical, given resources constraint, to have a medical doctor in every primary health care facility. However, efforts should be geared towards this through better planning, including forecasting, of the health worker force. In the interim, the need for upskilling of the available health workers is of utmost importance in providing the type of services that AYP will be willing to use [60].

It is likely that many SRH services such as contraceptive services will continue to be offered for free in many public health facilities in order to promote their uptake. Policymakers should be aware of both direct and indirect costs, or related costs such as costs for consumables which are often not covered in under free health services. Also sometimes, free health care has been associated with substandard care by users [61]. If free service is substandard, AYP will likely not use them as indicated by a willingness to pay for higher quality services.

Further research is needed to investigate scale heterogeneity [62], particularly differences among AYP across the country due to subnational differences in the social-ecological milieu and the lived experiences of AYP. Chandra-mouli et al. [63].

### Conclusion

The study generated new information on preference of AYP for SRH services. The preference for alternative care that has been observed may not represent true preference but a lack of options. Also, we found that the cadre of health workers attending to the adolescent and as well as the attitude of the health workers matters very much to AYP. Furthermore, we found that the cleanliness of the facility matters to the AYP, and that AYP were willing to spend more money to have a shorter waiting time. However, they are equally sensitive to time and cost. They are less likely to use a service if there is a long waiting time. Finally, adolescents and young people are not a monolithic group, and their personal characteristics determine their preferences. The factors associated with the preferences of AYP include their level of education and perceived socioeconomic status. Targeting a few key areas of service design and delivery may result in improvements in service acceptability and utilization. These results strengthen the call for involving adolescents in decision-making in health interventions for them and developing context-specific SRH programs for AYP in public health facilities.

### Supplementary Information

**Supplementary Material 1. **

**Supplementary Material 2. **

## Data Availability

Availability of data and materials The datasets used and/or analyzed during the study are available from the corresponding author on reasonable request.

## Abbreviations

AIC: Akaike information criterion
ASC: Alternative specific constants
ASRH: Adolescent sexual and reproductive health
AVC: Asymptotic variance–covariance
AYP: Adolescents and young people
BIC: Bayesian information Criteria
DCE: Discrete choice experiment
FGD: Focus group discussion
LCA: Latent class analysis
LGA: Local government area
LLR: Log-likelihood ratio
SRH: Sexual and reproductive health
WTP: Willingness to pay
