# Supplementary material for: Preferences in adolescents and young people’s sexual and reproductive health services in Nigeria: a discrete choice experiment
Source: Health Econ Rev. 2024 Mar 22;14:24. doi: 10.1186/s13561-024-00497-4 (PMC10958931; doi:10.1186/s13561-024-00497-4)
Supplement: Supplementary file 1 — Supplementary Material 1. [file 13561_2024_497_MOESM1_ESM.docx]

# **Supplementary document 1**

**Table 1: Cross tabulation of choice with sociodemographic characteristics, and SRH history of respondents**

|  | Health Facility 1 | Health Facility 2 | Neither | Total | Test of assoication |
| --- | --- | --- | --- | --- | --- |
|  | Freq (%) | Freq (%) | Freq (%) | Freq (%) |  |
| **Local Government Area** |  |  |  |  | 𝜒2(2) = 13.4792  **p=0.001** |
| Abeokuta South LGA | 1794 (51.9) | 1488 (43.1) | 174 (5.0) | 3456 (100.0) |  |
| Ijebu East LGA | 1788 (52.3) | 1516 (44.4) | 112 (3.3) | 3416 (100.0) |  |
| **Sex** |  |  |  |  | 𝜒2(2) = 1.8921  p=0.388 |
| Female | 1809 (52.6) | 1480 (43.0) | 151 (4.4) | 3440 (100.0) |  |
| Male | 1773 (51.7) | 1524 (44.4) | 135 (3.9) | 3432 (100.0) |  |
| **Age group** |  |  |  |  | 𝜒2(2) = 0.2267  p=0.893 |
| 15-19 years | 2460 (52.2) | 2053 (43.6) | 199 (4.2) | 4712 (100.0) |  |
| 20-24 years | 1122 (51.9) | 951 (44.0) | 87 (4.0) | 2160 (100.0) |  |
| **Occupation** |  |  |  |  | 𝜒2(4) = 17.8247  **p=0.001** |
| Student | 1971 (53.6) | 1572 (42.7) | 137 (3.7) | 3680 (100.0) |  |
| Apprentice/Artisan | 1100 (51.7) | 943 (44.3) | 85 (4.0) | 2128 (100.0) |  |
| Others | 511 (48.0) | 489 (46.0) | 64 (6.0) | 1064 (100.0) |  |
| **Educatonal status** |  |  |  |  | 𝜒2(6) = 34.2838  **p<0.001** |
| None | 110 (50.9) | 100 (46.3) | 6 (2.8) | 216 (100.0) |  |
| Primary | 408 (49.0) | 371 (44.6) | 53 (6.4) | 832 (100.0) |  |
| Secondary | 2879 (52.8) | 2381 (43.6) | 196 (3.6) | 5456 (100.0) |  |
| Higher | 185 (50.3) | 152 (41.3) | 31 (8.4) | 368 (100.0) |  |
| **Marital Status** |  |  |  |  | 𝜒2(4) = 7.2871  p=0.121 |
| Married | 209 (48.4) | 202 (46.8) | 21 (4.9) | 432 (100.0) |  |
| Divorced/Separated | 22 (68.8) | 8 (25.0) | 2 (6.3) | 32 (100.0) |  |
| Never married | 3351 (52.3) | 2794 (43.6) | 263 (4.1) | 6408 (100.0) |  |
| **Religion** |  |  |  |  | 𝜒2(8) = 8.6236  p=0.375 |
| Islam | 1198 (51.3) | 1039 (44.5) | 99 (4.2) | 2336 (100.0) |  |
| Orthodox (Non Catholic Christian) | 646 (54.9) | 485 (41.2) | 45 (3.8) | 1176 (100.0) |  |
| Catholic Christian | 224 (53.8) | 169 (40.6) | 23 (5.5) | 416 (100.0) |  |
| Pentecostal Christian | 1381 (51.4) | 1200 (44.6) | 107 (4.0) | 2688 (100.0) |  |
| Others | 133 (52.0) | 111 (43.4) | 12 (4.7) | 256 (100.0) |  |
| **Ethnicity** |  |  |  |  | 𝜒2(4) = 5.4685  p=0.243 |
| Igbo | 224 (52.8) | 174 (41.0) | 26 (6.1) | 424 (100.0) |  |
| Yoruba | 3164 (52.2) | 2655 (43.8) | 245 (4.0) | 6064 (100.0) |  |
| Others specify | 194 (50.5) | 175 (45.6) | 15 (3.9) | 384 (100.0) |  |
| **Subj socioeconomic standing (terciles)** |  |  |  |  | 𝜒2(4) = 36.8555  **p<0.001** |
| Lower | 1310 (50.5) | 1196 (46.1) | 86 (3.3) | 2592 (100.0) |  |
| Middle | 1614 (52.1) | 1359 (43.9) | 123 (4.0) | 3096 (100.0) |  |
| Higher | 658 (55.6) | 449 (37.9) | 77 (6.5) | 1184 (100.0) |  |
| Total | 3582 (52.1) | 3004 (43.7) | 286 (4.2) | 6872 (100.0) |  |
| **Ever used a primary or secondary health facility** |  |  |  |  | 𝜒2(2) = 0.6335  p=0.729 |
| No | 1479 (52.1) | 1249 (44.0) | 112 (3.9) | 2840 (100.0) |  |
| Yes | 2103 (52.2) | 1755 (43.5) | 174 (4.3) | 4032 (100.0) |  |
| Total | 3582 (52.1) | 3004 (43.7) | 286 (4.2) | 6872 (100.0) |  |
| **Risky sexual behaviour** |  |  |  |  | 𝜒2(4) = 19.5377  **p<0.001** |
| Never had sex | 539 (51.8) | 471 (45.3) | 30 (2.9) | 1040 (100.0) |  |
| Didn't use FP in last sexual encounter | 2199 (53.4) | 1761 (42.7) | 160 (3.9) | 4120 (100.0) |  |
| Used FP | 844 (49.3) | 772 (45.1) | 96 (5.6) | 1712 (100.0) |  |
| **Ever had STI** |  |  |  |  | 𝜒2(2) = 4.8688  p=0.088 |
| No | 3262 (52.0) | 2758 (44.0) | 252 (4.0) | 6272 (100.0) |  |
| Yes | 320 (53.3) | 246 (41.0) | 34 (5.7) | 600 (100.0) |  |
| **N** | **3582 (52.1)** | **3004 (43.7)** | **286 (4.2)** | **6872 (100.0)** |  |

**Table 2: Mixed logit model with cost as categorical variable**

| **Attributes and levels** | **β** | **Robust SE** | **p-value** |
| --- | --- | --- | --- |
| **Type of Staff** |  |  |  |
| Other health worker* |  |  |  |
| Doctor | 0.481 | 0.049 | <0.001 |
| Nurse | 0.333 | 0.037 | <0.001 |
| **Environment** |  |  |  |
| Not clean at all (1 star)* |  |  |  |
| Moderately clean (3 star) | 0.381 | 0.045 | <0.001 |
| Very clean (5 star) | 0.575 | 0.040 | <0.001 |
| **Health worker attitude** |  |  |  |
| Service provider is stern and may be judgmental* |  |  |  |
| Service provider is open and friendly | 0.187 | 0.032 | <0.001 |
| **Cost** |  |  |  |
| No cost* |  |  |  |
| N500 | -0.170 | 0.046 | 0.015 |
| N1500 | 0.177 | 0.067 | 0.022 |
| N2500 | -0.235 | 0.087 | <0.001 |
| **Time** | -0.072 | 0.035 | 0.041 |
| **Contraceptive** |  |  |  |
| May not always be available* |  |  |  |
| At least one method is always available | 0.141 | 0.031 | <0.001 |
| **Opening hours** |  |  |  |
| Weekdays only* |  |  |  |
| Weekdays and weekends | 0.050 | 0.040 | 0.207 |
| Weekdays and after hours | -0.022 | 0.037 | 0.556 |
| **Health facility ASC** |  |  |  |
| Health Facility 1 | 1.867 | 0.094 | <0.001 |
| Health Facility 2 | 1.770 | 0.095 | <0.001 |
| Neither* |  |  |  |

**Table 3: Likelihood ratio, CAIC and BIC for latent class logit models that achieved convergence**

| **No. of Classes** | **LLF** | **No of parameters** | **CAIC** | **BIC** |
| --- | --- | --- | --- | --- |
| 2 | -5314.69 | 35 | 10900.84 | 10865.84 |
| 3 | -5195.20 | 58 | 10840.22 | 10782.22 |
| 4 | -5122.03 | 81 | 10872.27 | 10791.27 |

*CAIC: consistent Akaike information criterion*

*BIC: Bayesian information criterion*
