# Supplementary material for: Preferences in adolescents and young people’s sexual and reproductive health services in Nigeria: a discrete choice experiment
Source: Health Econ Rev. 2024 Mar 22;14:24. doi: 10.1186/s13561-024-00497-4 (PMC10958931; doi:10.1186/s13561-024-00497-4)
Supplement: Supplementary file 2 — Supplementary Material 2. [file 13561_2024_497_MOESM2_ESM.docx]

**Supplementary document 2**

**Preferences in adolescent and young people’s sexual and reproductive health services in Nigeria: A discrete choice experiment**

**DCE quality checklist**

By our estimation, the study on preferences in adolescent and young people’s sexual and reproductive health services in Nigeria meets the criteria/checklist by Bridges et al [1], Hauber et al [2], Mandeville et al [3], and Lancsar & Louviere [4] to assess the validity of discrete choice experiment (DCE) studies. Firstly, the choice of attributes and levels was derived primarily from qualitative work with the target population in order to ensure comprehension and engagement with the choice tasks. Then, the attributes were selected such that they were conceptually distinct. All our attributes were unidimensional in order to ensure completeness and transitivity of our set of attributes and levels, as well as to minimize variability in their interpretation by study participants. We also included an opt-out option to mimic a real-life situation since participants would usually have the option of not using the services we investigated. We used an efficient experimental design based on informative (Bayesian) priors which capture maximum information for the model as such enabling reliable parameter estimation and more accurate preference estimates. We piloted our study tool amongst our target population. Although we did not acquire a sampling frame, our multilevel sampling approach from local government area (LGA), to the Ward, to the community, and household levels allowed for some randomness, and representativeness of the study population in our study sample. Data was collected by face-to-face interview and we continued to interview participants till the desired sample size of the study was achieved. We conducted pooled analysis for our study but we explored preference heterogeneity by running a model that included case-specific variables as well as a latent class logit model. We did not further explore scale heterogeneity since the study population was not fundamentally heterogeneous. The econometric model we used accounted for the panel nature of data in order to avoid the overestimation of the differences between preferences. Finally, we estimated a willingness-to-pay space model to allow for direct comparison among different attributes. Table 1 below contains a full assessment of quality checklist for our DCE study.

Table 1: Harmonized DCE quality checklist for *“Preferences in adolescent and young people’s sexual and reproductive health services in Nigeria: A discrete choice experiment”*

| **Quality Check** | **Response** |
| --- | --- |
| 1. **Well-defined research question is stated and is DCE is an appropriate method** |  |
| 1. Were a well-defined research question and a testable hypothesis articulated? | Yes; We hypothesized that sexual and reproductive health (AYP) make trade-offs based on quality of care characteristics in their choice to uptake sexual and reproductive health (SRH) services in public health facilities, and that they exhibit preference heterogeneity with respect to these services. |
| 1. Was the study perspective described, and was the study placed in a particular decision-making or policy context? | The study perspective was that of the participants and the policy context was the introduction of SRH services for AYP in public health facilities in Ogun State , Nigeria |
| 1. What is the rationale for using DCE to answer the research question? | Quantifying of tradeoff AYP were willing to making with respect to quality of facility-based SRH services |
| 1. **Attribute selection** |  |
| 1. Was attribute identification supported by evidence (literature reviews, focus groups, or other scientific methods)? How were they derived and validated? | Derived primarily from qualitative work with the target population |
| 1. Was the number of attributes appropriate? Was attribute selection justified and consistent with theory? | Yes; seven attributes were presented. There were aligned with the National standard and minimum package for adolescent healthcare in Nigeria [5]. |
| 1. Was the coverage appropriate? | Yes |
| 1. What form was used: generic or alternative specific? | Alternative specific |
| 1. Was price included? If so, was an appropriate payment vehicle used? | Yes |
| 1. Was risk included? If so, was it appropriately communicated? | Risk was not included in this study |
| 1. **Level selection** |  |
| 1. How were they derived and validated? Was level selection for each attribute justified by the evidence and consistent with the study perspective and hypothesis? | The process of attribute and levels development is published as a separate paper [6] |
| 1. Was the number of levels per attribute appropriate? | Two – four levels for the attributes |
| 1. Was an appropriate range used? | Yes |
| 1. Were the levels evenly spaced? | The levels are evenly spaced |
| 1. **Conceptualizing the choice** |  |
| 1. Was an unlabeled or labelled choice used? | The choice is unlabeled |
| 1. Was an opt-out, neither or status quo option included? | Opt-out options was used |
| 1. If a forced choice was used, was a justification provided? | Forced choice was used to capture participants who would not choose either of the health facility options |
| 1. Was the task incentive compatible? | Yes; participants were asked to choose a preferred health facility to obtaining sexual and reproductive health services based on the attribute-levels presented. |
| 1. **Experimental design** |  |
| 1. What type of design was used? Full factorial? Fractional factorial? If fractional, which effects are identified: main effects; main effects + higher order interactions? Was the choice of experimental design justified? Were alternative experimental designs considered? | D-efficient design was used for pilot study and D-efficient design with Bayesian priors was used for the main study |
| 1. How were the profiles generated and allocated to choice sets? | The profiles were generated using the Ngene software |
| 1. What are the properties of the design? Were the properties of the experimental design evaluated? | We used 24 choice tasks in three blocks which were designed to avoid dominated or duplicated alternatives, dividing the 24 choice tasks into three blocks to avoid respondents’ cognitive overload |
| 1. What is the efficiency of the design? | We used D-efficient design without attribute level balance using Bayesian priors. The D error is 0.02956±0.0006 |
| 1. Was identification checked (e.g. is the variance-co-variance matrix block diagonal)? Was the design blocked into versions? If so, how were choice sets allocated to versions? Were the resulting properties of the versions checked? | The design was blocked into three and choice set were automatically allocated by the Ngene software |
| 1. Were respondents randomly allocated to versions? How many choice sets were considered per respondent? | Multistage sampling was done to select participants; random sampling was done at each stage. However, blocks were assigned to communities and not individuals. Individuals in each selected community responded to the same set of choice tasks |
| 1. If some profiles were implausible – how was implausibility defined and how was it addressed? | None of the profiles were found to be implausible. Furthermore, Ngene allows for the removal of dominance during the experiment set up |
| 1. **Questionnaire design** |  |
| 1. Was an appropriate level of background and contextual information provided? Were the task instructions appropriate? | Task instructions and background information were provided. Sample Choice task was also provided |
| 1. Were the attributes and levels defined, and was any contextual information provided? | The attributes and levels were defined in the instructions |
| 1. Was the medium used to communicate attribute/level information (e.g. words, pictures, multi-media) appropriate? | Only text was used to communicate the attribute/level information. However it was interview facilitated in which interviewer and participants went through the choice task together on a tablet device |
| 1. Was the level of burden of the data-collection instrument appropriate? Was the number of DCE tasks included in the data-collection instrument appropriate? Were respondents encouraged and motivated? | Each participant completed 8 choice tasks apart from other accompany questions in the questionnaire for the study. Questionnaire was interviewer-administered |
| 1. **Piloting** |  |
| 1. Was coverage of attributes and levels checked? | Yes |
| 1. Was understanding and complexity checked? | Yes |
| 1. Was the length and timing checked? | Yes; Each interview was about 45 minutes |
| 1. **Sample and sample size** |  |
| 1. Were inclusion/exclusion criteria explicit? | Yes |
| 1. Was sample size appropriate for model estimation? | Sample size formula by de Bekker et al. [7] was used |
| 1. **Data collection** |  |
| 1. What recruitment method was used? | Recruitment of participants was done through a household survey |
| 1. How were data collected (e.g. mail, personal interview, web survey)? | Data was collected by face-to-face interview |
| 1. What was the response rate? | Sampling was done till study sample size was reached |
| 1. Were incentives used to enhance response rates? | No |
| 1. Was the sampling strategy justified (for example, sample size, stratification, and recruitment)? | Multistage sampling was done with random sampling in each stage |
| 1. Was the mode of administration justified and appropriate (for example, face-to-face, pen-and-paper, web-based)? | Tablet devices were used for data collection |
| 1. Were ethical considerations addressed (for example, recruitment, information and/or consent, compensation)? | Written informed consent was obtained from all participants after explaining study procedure, participants could opt out at any point without any consequences |
| 1. **Estimates: Choice of parameter estimates resulting from the model** |  |
| 1. Whether each variable corresponds to an effects-coded level, a dummy-coded level, or a continuous change in levels | All variables were dummy-coded level, apart from cost and waiting time that had a continuous change in levels |
| 1. Whether each variable corresponds to a main effect or interaction effect | Each variable corresponds to a main effect and no interaction effect were include in the estimate |
| 1. Whether continuous variables are linear or have an alternative functional form | Continues variables were assumed to be linear |
| 1. **Transparent description of the study** |  |
| 1. The data setup, including handling missing data | Data was set up as panel dataset, |
| 1. The software used for estimation | Stata v 16 was used for data analysis |
| 1. **Econometric analysis : Were statistical analyses and model estimations appropriate?** |  |
| 1. Were respondent characteristics examined and tested? | Yes |
| 1. Was the quality of the responses examined (for example, rationality, validity, reliability)? | A validity question was included |
| 1. Was model estimation conducted appropriately? Were issues of clustering and subgroups handled appropriately? | We conducted pooled analysis for our study but we explored preference heterogeneity by running a model that included case-specific variables as well as a latent class logit model. We did not further explore scale heterogeneity since the study population was not fundamentally heterogeneous. |
| 1. Were the estimation methods appropriate given experimental design and type of choice response? | Yes; Panel-data mixed logit models using random coefﬁcients to model the correlation of choices across alternatives in order to take preference heterogeneity into account, by the assumption that preferences in different attributes are independent |
| 1. Were alternative specific constants included? | Yes |
| 1. Was goodness of fit considered? | Information criteria (AIC and BIC) was used to discriminate between alternative models |
| 1. Sensitivity analysis of the model specification | Sensitivity analysis was done through predictive analysis |
| 1. Consistency of results estimated using different methods | Results were consistent using different methods |
| 1. Why the method was selected over alternative methods | Alternative methods will not account for the panel structure of the data |
| 1. **Assumptions of the model and the implications of the assumptions for interpreting the results** |  |
| 1. Assumptions about the error distribution | The error terms were assumed to be independent and identically distributed (IID) |
| 1. Assumptions about the independence of observations | The data has a panel structure with same respondent providing multiple outcomes, hence the observations from the same respondents are not independent |
| 1. Assumptions about the functional form of the value function | The functional form was assumed to be linear and continuous |
| 1. **Stochastic properties of the analysis** |  |
| 1. The statistical distributions of parameter estimates | We assumed that there was a normal distribution of preference weights across the sample |
| 1. The distribution of parameter estimates across the sample (preference heterogeneity) | Preference heterogeneity which was assumed to follow a multivariate normal distribution was assessed in this study |
| 1. The variance of the estimation function, including systematic differences in variance across observations (scale heterogeneity) | Scale heterogeneity was not assessed in this study |
| 1. **Trade-offs that can be inferred from the model** |  |
| 1. The magnitude and direction of the attribute-level coefficients | The magnitude and direction of coefficients are appropriate e.g. cost and waiting time had negative coefficients |
| 1. The relative importance of each attribute over the range of levels included in the experiment | This was assessed by expressing the preference coefficients in a willingness-to-pay space |
| 1. The rate at which respondents are willing to trade off among the attributes (marginal rate of substitution) | The relative differences in the WTP and probabilities of choosing an alternative helped to illustrate the trade-off that AYP make in their choice of SRH services. |
| 1. **Validity** |  |
| 1. Was internal or external validity investigated? | Internal validity was assessed by including a dominant choice task as an additional question |
| 1. Were answers for any respondents deleted and if so on what basis? | No responses were deleted |
| 1. **Were the results and conclusions valid?** |  |
| 1. Did study results reflect testable hypotheses and account for statistical uncertainty? | Yes |
| 1. Were study conclusions supported by the evidence and compared with existing findings in the literature? | Findings of study are supported by findings from literature |
| 1. Were study limitations and generalizability adequately discussed? | Study limitation and generalizability were discussed |
| 1. **Interpretation of the results taking into account the properties of the statistical model** |  |
| 1. Was the interpretation appropriate given coding of data? | Yes; categorical variables were dummy coded with the most negative expected level defined as the reference category |
| 1. Were results in line with a priori expectations? | Yes; e.g. participants were generally cost and waiting time averse. |
| 1. Were relative attribute effects compared using a common and comparable metric? | A model was estimated in the willingness to pay space that expressed all coefficients in monetary terms |
| 1. Conclusions that can be drawn directly from the results | Conclusions presented were drawn directly from the results |
| 1. Applicability of the sample, including subgroups or segments, to the population of interest | Latent class logit model allowed showed that the population of AYP was monolithic |
| 1. **Welfare and policy analysis** |  |
| 1. Was willingness to pay estimated using welfare theoretic compensating variation? | Yes |
| 1. Was probability analysis undertaken? | Yes |
| 1. Were marginal rates of substitution calculated? | Yes |
| 1. **Study presentation clear, concise, and complete** |  |
| 1. Was study importance and research context adequately motivated? | Yes |
| 1. Were the study data-collection instrument and methods described? | Yes |
| 1. Were the study implications clearly stated and understandable to a wide audience? | Yes |

# References

1. Bridges JFP, Hauber AB, Marshall D, Lloyd A, Prosser LA, Regier DA, et al. Conjoint Analysis Applications in Health — a Checklist : A Report of the ISPOR Good Research Practices for Conjoint Analysis Task Force. JVAL. 2011;14: 403–413. doi:10.1016/j.jval.2010.11.013

2. Hauber AB, González JM, Groothuis-oudshoorn CGM, Prior T, Marshall DA, Cunningham C, et al. ISPOR Task Force Report Statistical Methods for the Analysis of Discrete Choice Experiments : A Report of the ISPOR Conjoint Analysis Good Research Practices Task Force. Value Heal. 2016;19: 300–315. doi:10.1016/j.jval.2016.04.004

3. Mandeville KL, Lagarde M, Hanson K. The use of discrete choice experiments to inform health workforce policy: A systematic review. BMC Health Serv Res. 2014;14: 1–14. doi:10.1186/1472-6963-14-367

4. Lancsar E, Louviere J. Conducting discrete choice experiments to Inform Healthcare Decision Making A User ’ s Guide. Pharmacoeconomics. 2008;26: 661–677.

5. FMOH. National Standard and Minimum Package for Adolescent Healthcare. Abuja, Nigeria: Federal Ministry of Health; 2018.

6. Arije O, Madan J, Hlungwani T. Attributes development for a discrete choice experiment on preferences in sexual and reproductive health services for adolescents and young people in Nigeria. BMC Health Serv Res. 2022;22: 1511. doi:10.1186/s12913-022-08888-1

7. Bekker-grob EW, Donkers B, Jonker MF, Stolk EA. Sample Size Requirements for Discrete-Choice Experiments in Healthcare : a Practical Guide. Patient - Patient-Centered Outcomes Res. 2015;8: 373–384. doi:10.1007/s40271-015-0118-z
